# When participants get involved: reconsidering patient and public involvement in clinical trials at the MRC Clinical Trials Unit at UCL

**DOI:** 10.1186/s13063-018-2471-4

**Published:** 2018-02-07

**Authors:** Claire L. Vale, William J. Cragg, Ben Cromarty, Bec Hanley, Annabelle South, Richard Stephens, Kate Sturgeon, Mitzy Gafos

**Affiliations:** 1 PPI Group, MRC Clinical Trials Unit at UCL, 90 High Holborn, London, WC1V 6LJ UK; 2 Department of Global Health and Development, Faculty of Public Health and Policy, London School of Hygiene and Tropical Medicine, Keppel Street, London, WC1E 7HT UK

## Abstract

**Background:**

Patient and public involvement (PPI) in clinical trials aims to ensure that research is carried out collaboratively with patients and/or members of the public. However, current guidance on involving clinical trial participants in PPI activities is not consistent.

**Methods:**

We reviewed the concept of participant involvement, based on our experience. Two workshops were held at the MRCCTU at UCL with the aim of defining participant involvement, considering its rationale; benefits and challenges; and identifying appropriate models for participant involvement in clinical trials. We considered how participant involvement might complement the involvement of other public contributors. Both workshops were attended by two patient representatives and seven staff members with experience of PPI in trials. Two of the staff members had also been involved in studies that had actively involved participants. They shared details of that work to inform discussions.

**Results:**

We defined trial participants as individuals taking part in the study in question, including those who had already completed their trial treatment and/or follow-up. Because of their direct experience, involving participants may offer advantages over other public contributors; for example, in studies of new interventions or procedures, and where it is hard to identify or reach patient or community groups that include or speak for the study population.

Participant involvement is possible at all stages of a trial; however, because there are no participants to involve during the design stage of a trial, prior to enrolment, participant involvement should complement and not replace involvement of PPI stakeholders. A range of models, including those with managerial, oversight or responsive roles are appropriate for involving participants; however, involvement in data safety and monitoring committees may not be appropriate where there is a potential risk of unblinding.

Involvement of participants can improve the trial experience for other participants; optimising study procedures, improving communications; however, there are some specific, notably, managing participant confidentiality and practicalities relating to payments.

**Conclusions:**

Participant involvement in clinical trials is feasible and complements other forms of PPI in clinical trials. Involving active participants offers significant advantages, particularly in circumstances where trials are assessing new, or otherwise unavailable, therapies or processes. We recommend that current guidance on PPI should be updated to routinely consider including participants as valid stakeholders in PPI and potentially useful approach to PPI.

## Background

Patient and public involvement (PPI) describes a variety of activities that aim to ensure that trials are carried out collaboratively with patients and/or members of the public. There are a number of arguments to support PPI in healthcare research, including clinical trials. For example, there is a case that the public have a right to be involved in publicly funded research that may impact on their own health, or the services that they receive [1], with many funding bodies now encouraging or insisting upon some form of PPI in the trials that they fund. It has also been argued that the experiences and personal insights of patients, carers and service users can help to improve research quality, relevance and impact [2]. On a practical level, reports have implied that PPI improves the overall quality of clinical trials and increases the likelihood that they will be completed successfully [3–6].

Despite evidence of increased PPI in health research, including clinical trials, in recent years [7, 8], little is published about involvement of trial participants. The current INVOLVE definition (http://www.invo.org.uk/find-out-more/what-is-public-involvement-in-research/) of public involvement in health and social care research excludes research participants from its classification of members of the public. It states that for most studies, ‘it is not appropriate for people involved in the research to also be participants in the research as that can compromise both the researcher and the person involved,’ with the possible exceptions of participatory or action research studies (http://www.invo.org.uk/posttyperesource/how-to-find-people-to-involve/). In contrast, other organisations have highlighted circumstances when it may not only be possible, but also desirable, to involve participants. For example, the UNAIDS Good Participatory Practice (GPP) guidelines for biomedical HIV-prevention trials [9] and similar guidance for tuberculosis (TB) trials [10], provide trialists with guidance on how to involve a range of stakeholders, including trial participants, in the design and conduct of clinical trials.

In a recent study of PPI in clinical trials conducted by the Medical Research Council Clinical Trials Unit (MRCCTU) at University College London (UCL), we identified three studies that had actively involved participants [11] including two randomised controlled trials of HIV prevention, set in the UK [12] and Africa [13], and one cohort study of young people in the UK [14]. In the two randomised trials, participants were actively involved as collaborators or partners, offering advice to researchers on the conduct of the studies, based on the unique experiences and insights that participating in the study offered. As we were unaware of any other reports in the health literature relating to active involvement of trial participants in PPI and because existing advice to researchers about involving participants was conflicting, we have explored participant involvement as a distinct component of PPI activities in clinical trials. We aimed to highlight inconsistencies between existing guidance on participant involvement in PPI strategies for clinical trials, to provide guidance and influence researchers to give due consideration to this potentially novel aspect of PPI for future trials.

## Methods

The MRCCTU PPI Group is the Steering Committee responsible for developing, promoting and supporting the active involvement of patients and the public in all clinical studies that are being led by the MRCCTU. In August and October 2015, nine members of the PPI steering group (including two patient representatives, who work closely with the MRCCTU and with other organisations, six MRCCTU staff members, and an independent specialist advisor) attended two discussion workshops on the active involvement of participants. All the staff members have experience of PPI in their research roles, either in clinical trials, cohort studies or systematic reviews, and two of the staff members had been directly involved in the three studies that had actively involved participants. The patient representatives had been actively involved in PPI in various clinical trials and other research, although neither had experience of being actively involved in PPI of trials in which they were also participating. The workshops were chaired by the independent specialist advisor.

During the discussion workshops, we focussed on defining:

- Trial participants, as distinct from patients and members of the public
- Active participant involvement
- The rationale for involving participants
- When to involve participants in PPI
- How to involve participants in PPI
- Challenges specific to participant involvement

Although we aimed to reach consensus among committee members at the workshops on each of these topics, given the small number of members and limited number of trials in which participant involvement had been implemented, this was intended to be exploratory and informative. No formal approach to reaching consensus (e.g. a Delphi process) was undertaken, nor would it have been appropriate. On each point of the workshop discussions the chair facilitated discussions between the members and recorded the agreed points. At the second workshop, these were revisited and if needed and appropriate, revised, such that the final report from the two workshops was agreed by all members.

This paper summarises the findings of the workshops, providing précis of participant involvement in two clinical trials at the MRCCTU at UCL.

## Results: workshop discussions and consensus

### Defining trial participants

For the purposes of these discussions, we defined active trial participants as an individual taking part in the trial in question, irrespective of what stage the trial has reached. We aimed to distinguish between these ‘active’ participants and other potential PPI contributors (Table 1). For example, ‘active participants’ might include individuals who:

**Table 1:** Definitions of participants and other public contributors

	Participant	Patient/member of the public
Someone who is a participant in this trial, even if they are no longer actively receiving treatment or attending appointments	✓	X
Someone who is (or has been) a participant in another trial(s) or who may become a participant in the future	X	✓
Someone who participated in a feasibility or pilot study that led to this trial	X	✓
Someone who gave consent for an individual to participate in this trial, e.g. the parent of a child	✓	X
Carers, patients or people from organisations that represent patients, carers or other service users in this trial	X	✓

- Are still undergoing treatment or receiving an investigational product as part of the trial
- Are still actively attending a clinic for scheduled follow-up
- Are providing information to that study more passively; for example, where follow-up information is collected through a registry
- Have completed their own treatment or use of an investigational product and follow-up while recruitment of new participants to the trial is still continuing
- Are ‘proxy participants’; for example, in trials involving young children or infants, where parents or care givers provide consent on behalf of their child, the people providing consent are included in our definition of participants

Individuals who had participated in a previous trial, or who may participate in future trials were not considered to be ‘active participants’ in this context and, while they may still be involved in PPI activities as patient representatives or members of the public, we did not consider this to be participant involvement as we are describing here.

### Defining active participant involvement

Our definition of active participant involvement is based on INVOLVE’s broader definition of PPI. As such, we define active participant involvement in clinical trials as, ‘research being carried out “with” or “by” *trial participants*, rather than “to”, “about” or “for” them’. We consider involvement of participants to be one form of PPI in which trial participants are actively involved in contributing to the conduct of the study. We do not believe that involving participants should replace or preclude the involvement of other public or patient contributors to research, but that the approaches are complementary.

INVOLVE distinguishes between ‘involvement’ where ‘members of the public are actively involved in research projects and in research organisation’; ‘engagement’ where ‘information and knowledge about research is provided and disseminated’; and ‘participation’ where ‘people take part in a research study’. Although active involvement of trial participants requires both participation in, and engagement with, the clinical trial, it is important to be clear that while clinical trials regularly collect quantitative and qualitative data on participants’ experiences in trials, such activities are all examples of trial ‘participation’ and not ‘involvement’.

Table 2 aims to further clarify whether different activities within clinical trials are classified as involvement, engagement or participation, and further examples of involvement activities are detailed in Case study 1.

**Table 2:** Examples of active participant involvement, other patient and public involvement, engagement and participation in clinical trials

Activity	Active participant involvement	Patient/public involvement	Public engagement	Trial participation
Patients, carers and/or family members take part in discussions to plan a trial	X	✓	X	X
Trial participants take part in a discussion about the recruitment strategy or the development of a new questionnaire	✓	X	X	X
Trial participants take part in in-depth interviews about their experience of taking part in the trial	X	X	X	✓
Representatives (e.g. staff, patients or family members) from a patient organisation take part in a discussion about disseminating trial results	X	✓	X	X
Trial participants complete questionnaires about how the trial is conducted	X	X	X	✓
Results of the trial are presented to participants, patients and/or the public	X	X	✓	X

Case study 1. Participant involvement in practice: The PROUD trial

**Table:** 

PROUD was an open-label, wait-listed randomised controlled trial (RCT) which evaluated oral pre-exposure prophylaxis (PrEP) as a HIV-prevention option among gay men, other men who have sex with men and trans women attending sexual health clinics in England.
We developed an advertisement template on which to advertise participant involvement meetings. We submitted the template to the Ethics Committee for approval at the beginning of the trial and were able to add details of the specific meetings as required. In advance of a participant involvement meeting, the advertisement was distributed to participants in the clinics (by hand and email), was posted on the website which participants accessed to complete self-reported data, and was distributed via a central trial mailing list that participants could voluntarily sign up to. Participants who were interested in getting involved with the advertised activity would contact the PPI coordinator who would provide additional information.
We held participant involvement meetings in person, via teleconference and WebEx, or linked regional in-person meetings via video-conferencing facilities. Some topics were discussed in multiple meetings; for example, the PROUD study results were discussed at four participant involvement meetings in Brighton, London, Manchester and Sheffield.
We usually scheduled participant involvement meetings before Community Engagement Group (the CEG was a more traditional PPI advisory group) and Trial Steering Committee (TSC) meetings and shared reports of the participant involvement meetings with the CEG and TSC membership. PPI representatives on the TSC, CEG and Data Safety Monitoring Committees (DSMC) were usually invited to facilitate or attend the participant involvement meetings. In the interests of transparency, we also posted the reports of the meetings on our website (www.proud.mrc.ac.uk/patient_and_public_involvement).
In addition, we involved participants via email discussions; for example, asking for their involvement to review a new ‘end of study’ case report form (CRF) and review a new infographic explaining alternative adherence regimens.

### Defining the rationale for active participant involvement

Participants in a clinical trial have a direct and unique experience of both the issue under investigation and of taking part in the specific trial (Case study 2). As such, they can offer an additional perspective to that of the other public contributors. For example, their first-hand experience of the trial processes and procedures can be useful when revising recruitment, retention or adherence strategies. This may be particularly useful in trials of new interventions or procedures, where trial participants are the only people with experience of the intervention or procedure under investigation. Similarly, participant involvement may be more valuable in trials where other models of PPI might be difficult; for example, when it is hard to identify or reach the trial population or where organisations that include or speak for the trial population do not exist. One example may be a trial in people at risk of sexually transmitted infections, as was the case in both the PROUD and MDP301 trials.

Another potential advantage of involving participants may be in advocating for the implementation of the trial results. Patient representatives are regularly involved in defining key messages and communicating study results. In this context, trial participants can equally describe the relevance and implications of the intervention. This can be very powerful in communicating trial results and helping people understand the human implications. Although there may be trials in which involving participants may not be necessary, feasible or desirable in all trials, due consideration of whether and how participants might be involved in the PPI strategy should be made at the planning stages.

Case study 2. Rationale for participant involvement: The MDP301 trial

**Table:** 

The Microbicides Development Programme (MDP) 301 phase III, randomised, double-blind, placebo-controlled clinical trial evaluated the safety and effectiveness of a vaginal microbicide gel among HIV-negative women in East and Southern Africa. We invited participants to be involved in PPI activities throughout the life cycle of the trial. Participants provided a unique perspective to PPI in this trial for a number of reasons:
1. Participants had a unique experience of using a vaginal microbicide gel as a novel HIV-prevention technology which was not available outside of the trial 2. In an environment in which many formal structures were male dominated and women’s voices were often hidden or drowned out by socio-cultural norms of gender inequity, participant involvement facilitated an opportunity to hear women’s voices as part of the PPI process. It was difficult both to elicit input from women, and to involve women in a discussion about a novel prevention technology in male-dominated spaces. However, enrolment in the trial created safe spaces, in which women were more able to provide input, and were eager to be involved with PPI activities 3. Participants were HIV-negative volunteers, representing a population group that can be difficult to identify through other community sources such as HIV support charities and support groups 4. HIV was a stigmatised topic within these communities and it was especially difficult for married women to voice their opinions about the need to protect themselves from HIV, but women found it more acceptable to do so when in the exclusive company of other women enrolled in a HIV-prevention trial

### Defining when participants may be actively involved

**Fig. 1:**
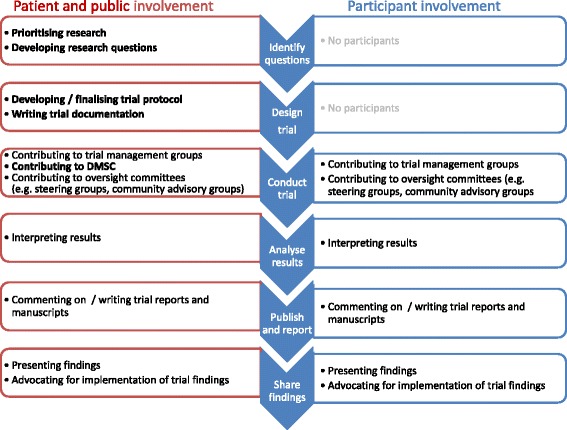
Participant involvement and patient and public involvement (PPI) at different stages of the clinical trial research cycle. A comparison of opportunities for patient and public contributors and active trial participants involved in PPI throughout the trial cycle

PPI has a role throughout the life cycle of a clinical trial, from identifying the research question to disseminating the research results. The role of participant involvement in the trial cycle mirrors other types of PPI at every stage (Fig. 1), with the notable exception of prior to the start of trial enrolment when there are no participants enrolled in the trial. However, at each remaining stage, participant involvement can have an additional or complementary role to other PPI.

### Defining how to actively involve participants

Participants can be invited to become involved with PPI in a number of ways; for example, via existing trial participant groups (for example, adherence support groups), by forming specific participant involvement groups, or by involving interested individuals directly.

The roles that participants may become involved in within a trial may be managerial, oversight or responsive roles [15], (Table 3). In our experience, while the involvement of trial participants on managerial and oversight committees is clearly defined, the concept of trial participants’ involvement in responsive PPI models is frequently confused with the concept of qualitative research. As such, it is important to stress the distinction. When involved in PPI, trial participants should be treated in the same way as other patient and public contributors and the questions posed to should be comparable. For example, if a trial is experiencing problems with recruitment, the trial team may arrange a PPI meeting, in which the involved participants, patients and public contributors work collaboratively with the researchers to devise strategies to improve the situation. This is very different from qualitative research, whereby a researcher may interview participants about their *experiences* of trial recruitment. The researcher then analyses the data and produces findings. As two distinct approaches, participant involvement does not replace the role of qualitative research and vice versa.

**Table 3:** Potential models of participant involvement in trials

Role	Model	Appropriate for participant involvement?	Comments
Managerial	Patient/public representative on Trial Management Group	Yes	Thought should be given to how to recruit and train participants who might be involved in a TMG – they need an understanding of research and to be confident and articulate in this type of setting
Oversight	Patient and participant research partners	Yes	A committee made up of participants and patients with an oversight remit and clear reporting route into TMG and/or TSC
	Patient/public representative on Trial Steering Committee	Yes – but with caveats	Should not be voting members as not independent of the trial
	Patient/public representative on Data Safety Monitoring Committee	No	Involvement not appropriate because of danger of un-blinding and of bias Information from an advisory group (e.g. patient and participant research partners) could be considered by a DSMC
Responsive	Involvement on specific tasks (e.g. facilitated through existing patient groups)	Yes	Participants involved in specific tasks, either on a one-off or ongoing basis. For example, participants help to design interview schedules, draft key messages of trial results, be advocates for the trial findings, etc.
	Ad hoc participant meetings	Yes	Participant meetings to discuss topics and issues as they arise. For example, ways to address slow accrual, ways to respond to negative rumours, etc.
	Ongoing participant groups	Yes	Regular meetings of participants to provide feedback and actions to trialists on a regular basis
	Community advisory groups	Yes	Participants contribute via membership of a community advisory group for the ongoing trial
	Community meetings to advise trial teams	Yes	Participants contribute via community meetings held to advise the ongoing trial

Using a range of PPI models to involve participants is likely to increase involvement from a greater diversity of participants. For example, some participants may prefer long-term commitment to trial oversight committees while others may prefer to be involved in responsive roles, such as ad hoc, one-off meetings, or providing input on issues or topics in which they are most interested (Case study 2).

There was only one PPI role identified as not being appropriate for trial participants and that was active involvement in DSMCs, particularly in blinded trials. This was due to the likelihood of DSMC members being unblinded and participants potentially finding out which arm of the trial they are randomised to. Careful consideration also needs to be given to participant involvement in PPI activities that could risk data contamination (e.g. where participants believe that they know what arm they are in) or exposure of hidden randomisations (such as in a Zelen design [16], where randomisation, but not the intervention, takes place prior to consent being agreed). In the latter example, instead of prohibiting participant involvement in PPI it may be a case of deferring participant involvement until after the occurrence of the hidden randomisation.

### Defining challenges of actively involving participants in PPI in clinical trials

As with other types of PPI, there are a number of challenges to involving participants that need to be considered; for example, considering that people with a disease or condition may become too sick to remain involved or that specific training or support for participants to become involved constructively may be needed. As for all PPI, trialists should consider additional requirements that actively involving participants may require and plan for this appropriately in terms of trial budget and resources. Similarly, as with other forms of PPI, the representativeness of the contributors may be called into question; therefore, it is important to be clear that the goal is to seek a broad range of *perspectives* rather than trying to gain representativeness of the population groups (http://www.invo.org.uk/posttyperesource/how-to-find-people-to-involve/).

There are some challenges that are unique to involving participants (Case study 3). One key challenge relates to participant confidentiality, and how best to manage that confidentiality in the context of needing to store personal information (names and contact details) in order to facilitate involvement. Related to this is the practical issue of paying or reimbursing participants for their time and travel costs, in line with other PPI stakeholders. This can potentially cause conflict between respecting confidentiality of trial participants and holding personal details in order to facilitate payments, but there is also the danger of creating the impression that the involved participants are being ‘paid’ (or receiving special consideration) and others not. There are also specific challenges around confidentiality where the populations involved may be susceptible to being stigmatised or discriminated against. Another challenge specific to the involvement of participants from a research design perspective is the risk of ‘involvement’ undermining blinding procedures or contamination between arms. Risks to the trial design need to be considered when deciding whether to involve participants in PPI and when choosing which models of involvement to employ.

An additional challenge specific to involving participants is that while PPI activities do not require ethical approval or consent from stakeholders, because participant involvement is still a new approach, it may be necessary to discuss requirements directly with ethics committees, making a clear distinction between materials inviting participants to take part in PPI and recruitment material for the trial to avoid potential confusion.

There are also specific challenges of involving participants from different populations in different contexts; for example, involving participants in countries with no tradition of PPI or with no culture of challenging medical advice, and involving participants who live distant to the research centres or who cannot easily travel to where the trial is being conducted. However, there are a range of alternative options to overcome some of these challenges, such as partnering participants with other PPI stakeholders and/or facilitating remote involvement instead of face-to-face meetings for example. However, these factors should only affect decisions on how to involve participants rather than whether to involve them.

Case study 3. Challenges of participant involvement: experiences from the PROUD trial

**Table:** 

In PROUD, the main challenges related to managing participant involvement within a clinical trial unit whose expectations are that trialists will not have direct contact with participants. As such, it was challenging setting up systems to hold participants’ email addresses, setting up the mailing list, and even registering participants by name for meeting attendance at the CTU offices. We overcame these challenges by largely managing direct email contact between the participants and the PPI coordinator via a secure and dedicated NHS email account, finding a secure one-way distribution mailing list, and creating a way to register participants by numbers instead of names for the meetings (proud1, proud2, etc.).
There were challenges in explaining participant involvement to other researchers and PPI stakeholders, and in distinguishing participant *involvement* from *participation* in qualitative research or participant *engagement* activities. However, there were no problems in explaining participant involvement to trial participants.
The Ethics Committee wanted to approve the template advertisement for meetings, but then agreed that the involvement processes were equivalent to other PPI activities and did not require additional ethics review.
PROUD was an open-label trial in which 50% of participants were not given any product for the first year of follow-up. The Trial Management Group discussed restricting participant involvement meetings to online or telephone meetings or holding separate meetings with participants in the active and control groups in order to avoid participants meeting and potentially sharing products. However, we already knew that participants could meet each other at the research clinics, we encouraged snowball recruitment, and our baseline data suggested social and sexual networking within the cohort. Consequently, we decided that face-to-face participant involvement meetings did not substantially increase the risk of contamination. However, the circumstances of each trial are different and research teams need to consider which PPI models best suit the purposes of their specific trials to avoid introducing risks to the trial design.

## Discussion

Active involvement of participants in PPI can benefit the delivery of clinical trials by bettering the trial experience for current and future trial participants, optimising study procedures, and improving communication of key messages and results. Participant involvement may complement other PPI activities relating to clinical trials, recognising the unique perspective of participants as one of a number of key stakeholders in PPI processes.

In the trials we have considered, we have demonstrated that including trial participants in PPI can add unique insight and input into the running of a trial in which they are participating. This may be particularly valuable in trials where the participants are not otherwise organised into established groups (for example, in prevention trials), or where there are cultural or societal barriers to more traditional PPI roles. It may also be valuable in trials of novel treatments in which trial participants are the first or only stakeholder with experience of the treatment. However, because this approach is novel to many researchers and PPI stakeholders, a number of challenges and some practicalities, as discussed here, need to be thoroughly considered.

Clearly, there are limitations of this work. As this is a novel and emerging approach, we are aware of only two clinical trials at the MRCCTU in which participants have actively contributed to PPI, although in line with the UNAIDS GPP guidelines this is common practice in HIV-prevention trials conducted globally. Our discussions were, therefore, greatly influenced by the limited sample in which there was a largely positive experience of participant involvement. We also acknowledge that we did not undertake a formal consensus-gathering approach as it would not have been appropriate in this context. That said, the authors represent a range of perspectives including researchers, with and without direct experiences of participant involvement, but all with experience in PPI, and from patient and public contributors with extensive clinical trial experience. All agreed on certain advantages of involving participants. We are not suggesting that all trials must have participant involvement, or that this type of involvement should replace the need for involvement of other PPI stakeholders. We see the approaches as complementary. There may well be trial scenarios that we have not considered where participant involvement would be unlikely to add extra benefits to PPI.

We aim to continue to evaluate the role of participants in PPI and to explore appropriate models to enhance their involvement. We are, therefore, conducting a formal evaluation of the experiences of participants and researchers in the PROUD study which we hope will add further weight to the narrative presented here on participant involvement as this field continues to emerge.

## Conclusions

The continuing evolution and development of PPI in clinical trials should include actively involving participants. We recommend that trialists and trials units give due consideration to actively involving trial participants in their trials, in particular where interventions under investigation are novel and where an obvious patient group is not well established. We also recommend that organisations such as INVOLVE reconsider their definitions of PPI stakeholders to ensure that trial participants are included within their remit.
